# Quantitative Genetic Assessment of Female Reproductive Traits in a Domesticated Pacific White Shrimp (*Penaeus vannamei*) Line in China

**DOI:** 10.1038/s41598-020-64597-x

**Published:** 2020-05-12

**Authors:** Shengjie Ren, Peter B. Mather, Peter Prentis, Yutao Li, Binguo Tang, David A. Hurwood

**Affiliations:** 1 Science and Engineering Faculty, Queensland University of Technology, Brisbane, QLD 4001 Australia; 2 Australian Rivers Institute, Griffith University, Brisbane, QLD 4111 Australia; 3 CSIRO Agriculture & Food, Brisbane, QLD 4067 Australia; 4 Beijing Shuishiji Biotechnology Co. Ltd., Beijing, 102600 P. R. China

**Keywords:** Agricultural genetics, Animal breeding

## Abstract

Seed production can be improved if genetic selection is applied to key reproductive traits when a substantial amount additive genetic variation is present that can be exploited in a selective breeding program. Despite the commercial importance of reproductive traits to the seed production sector currently, few quantitative genetic studies have been conducted to address these traits in farmed penaeid shrimp culture lines. Here, we investigated genetic parameters for a number of key reproductive traits that directly impact nauplii production in Pacific white shrimp (*P. vannamei*) hatcheries in China. Our objectives were to determine the additive genetic variance associated with reproductive traits, and to anticipate any potential impacts on reproductive performance when selecting for increased body weight by assessing genetic correlations between post-spawning body weight and specific female reproductive traits. Data were collected on 595 females from 78 full-sib families over 30 days, with a total of 1,113 spawning events recorded. Traits studied included: body weight after spawning (WAS), number of eggs per spawn (NE), number of nauplii per spawn (NN), egg hatching rate per spawn (HR), number of eggs produced relative to female weight (g) (FE), and spawn frequency over 30 days (SF). Estimated heritability was high  for WAS (*h*^2^ = 0.64 ± 0.10) and moderate for NE (0.26 ± 0.07), NN (0.18 ± 0.06), and SF (0.15 ± 0.06), respectively. In contrast, *h*^2^ for HR (0.04 ± 0.03) and FE (0.05 ± 0.04) were low. The genetic correlations between growth trait (WAS) with NE, NN and SF were 0.93 ± 0.10, 0.84 ± 0.10, and 0.57 ± 0.18, respectively. While the genetic correlation between WAS and HR was low (0.02 ± 0.33), a negative genetic correlation was found between WAS and FE (−0.50 ± 0.27). Overall, we concluded that it is possible to improve the key female reproductive traits (i.e. NE, NN, and SF) in cultured white shrimp lines via genetic selection, but not for HR or FE. The genetic relationship between the growth trait and reproductive traits predicts that selection on fast growth would increase the production in the seed sector, with little or no compromise on the eggs quality.

## Introduction

Reproductive characteristics constitute a set of commercially important traits that are yet to receive much attention when genetic improvement is applied to farmed aquatic species^1–3^. This is especially true for penaeid shrimp species that possess many unique reproductive characteristics; in particular, at maturation in hatcheries, many females may spawn relatively infrequently or may never spawn, while a small proportion of females spawn multiple times, hence these females are likely to contribute the majority of nauplii produced^4–6^. Having knowledge about genetic parameters (heritability and genetic correlations) for key reproductive traits will be essential when designing better breeding strategies for improving broodstock reproductive capacity and quality via genetic selection. For example, a recent experimental study of a small marine copepod crustacean (*Parvocalanus crassirostris*) reported that total egg production was increased by 24.5%, following selection over five generations with heritability (*h*^2^) for this trait estimated at 0.38^7^.

While application of genetic selection to farmed aquatic species has increased productivity significantly in a number of species^8,9^, most breeding programs to date have focused primarily on growth traits^2,10,11^. There are many examples of this practice however, that have also reported undesirable (cor)related effects on other fitness traits in domesticated animals, particularly with respect to metabolic, reproductive and health status traits^12^. For example, negative correlations were reported in pigs for reproductive traits when individuals were selected for high meat production efficiency. This resulted in a significant reduction in both fertility and litter size^12^. In contrast, studies in a wide variety of animal species (including model species such as mice, rabbits, dogs, sheep, pigs and salmon) have reported positive relationships between growth traits and some reproductive traits (e.g. litter size and fecundity)^13^. While the practice of genetic selection works essentially via a ‘black box’ approach, the opportunity still remains to understand, to anticipate and to prevent any potentially negative impacts of selection based on developing knowledge about genetic correlations (*r*_*g*_) between growth traits and important reproductive traits^12^.

To date, few studies have investigated genetic parameters for reproductive traits in farmed aquatic taxa, and of those studies, most have focussed on salmonid species^14–19^ and tilapias^20–22^. In salmonids, the majority of production traits that have been investigated show relatively high levels of additive genetic variance with most genetic correlations indicating positive relationships between growth-related traits and the reproductive traits examined. As an example, Gall and Neira^15^ reported *h*^2^ estimates for weight, number of green eggs, and number of eyed eggs in coho salmon (*Oncorhynchus kisutch*) ranging between 0.32 to 0.42, and genetic correlations between harvest weight and reproductive traits of egg weight and weight of eggs spawned were the same at 0.46. In domesticated rainbow trout (*Oncorhynchus mykiss*), *h*^2^ estimates for female egg number and egg size were 0.32 and 0.28, respectively^14^, while *h*^2^ estimates for traits including spawning date, egg size, number of eggs, and egg volume were all moderate to high^19^. In contrast in a Nile tilapia (*Oreochromis niloticus*) breeding program in Vietnam, *h*^2^ estimates for both fecundity traits and fertility were low^21^, while spawning success (*h*^2^ = 0.20–0.22) was an exception^22^. Likewise, Thoa *et al*.^20^ reported low *h*^2^ estimates for number of fry at hatching, total fry weight, and fry mortality in a base population of red tilapia (*Oreochromis* sp.). In addition, a case study that applied selection on reproductive traits in female channel catfish to produce hybrid catfish embryos reported realized *h*^2^ estimates for fecundity that ranged from 0.10 to 0.42 while *h*^2^ for percentage hatch and fry/kg were low^23^.

While a number of studies have demonstrated the potential capacity to improve seed production via genetic selection^4–6^, there has been a noticeable lack of research that has investigated capacity of females to spawn multiple times in penaeid shrimp. This trait has the potential to double or even triple nauplii production by individual females. Until recently however, there have only been a few reports that have investigated genetic parameters for this trait in penaeids^24,25^. Reproductive traits in farmed penaeid species (specifically fecundity-related traits), are among those that can contribute the most to increasing profitability of the hatchery sector but to date, only a single study has reported *h*^2^ estimates for the above traits under commercial hatchery conditions^26^. While there have been two studies estimating genetic parameters for reproductive traits in Pacific white shrimp (*P. vannamei*) following artificial insemination^27,28^, more information will be required for the seed production sector because these studies indicate that there are differences in reproductive trait data when applying artificial insemination vs natural mating in penaeid species. Moreover, high levels of additive genetic variance in reproductive traits for mean oocyte number, diameter, ovary maturity stage^29^, and high genetic correlations between levels of vitellogenin in haemolymph and mean diameter of oocytes have been reported for Pacific white shrimp^30^. In black tiger shrimp (*P. monodon*), *h*^2^ estimates for days to spawn, number of eggs, number of nauplii, and hatching rate ranged from 0.18 to 0.47, indicating that these traits can be improved to increase total reproductive output via genetic selection^31^.

The aims of the current study therefore were: (1) to examine genetic variance for key reproductive traits in female *P. vannamei* broodstock under commercial maturation conditions using the family line, and (2) to estimate genetic correlations between individual body weight after spawning with specific reproductive traits. Knowledge generated in this study can be applied to improve production of nauplii via genetic selection and to minimize any negative impacts on reproductive performance that may result from selection for fast growth.

## Results

### Descriptive statistics

Across a 30-day experimental period, 950 successful spawning records were observed out of a total of 1,113 spawning events. The basic statistics for each trait (number of observations, mean, minimum, maximum, standard deviation and coefficients of variation) are presented in Table 1. Mean body weight after spawning (WAS) was 39.66 ± 8.44 g (ranging from 18.19 to 70.63 g). Mean number of eggs (NE, 225.15 × 10^3^) and number of nauplii (NN, 194.63 × 10^3^) per spawn were in general terms, comparable with that obtained during commercial Pacific white shrimp nauplii production elsewhere in China (pers. obs.). A relatively high mean hatch rate (HR = 84.60%) estimate here indicates that the experimental broodstock management protocols employed had been appropriate.

**Table 1:** Descriptive statistics of reproductive traits for female *Penaeus vannamei*.

Trait	Unit	N	Mean	Minimum	Maximum	Standard deviation	Coefficient variation (%)
WAS	g	947	39.66	18.19	70.63	8.44	21.27
NE	×10^3^	949	225.15	33.00	677.25	79.97	35.52
NN	×10^3^	949	194.63	0.00	616.50	91.94	47.24
HR	%	950	84.60	0.00	1.00	0.23	27.57
HR^at^	unit	950	69.83	0.00	90.00	17.99	25.76
FE	×10^3^/g	946	5.74	1.35	12.80	1.82	31.76
SF	unit	595	1.44	0.00	6.00	1.34	93.40

WAS = Weight after spawning, NE = Number of eggs per spawning, NN = Number of nauplii per spawning, HR = Fertilization rate %, HR^at^ = Arcsine transformed of HR, FE = Number of eggs per unit weight, SF = Number of spawns during experimental period of 30 days.

### Phenotypic relationships between body weight and number of eggs/nauplii per spawn

**Figure 1:**
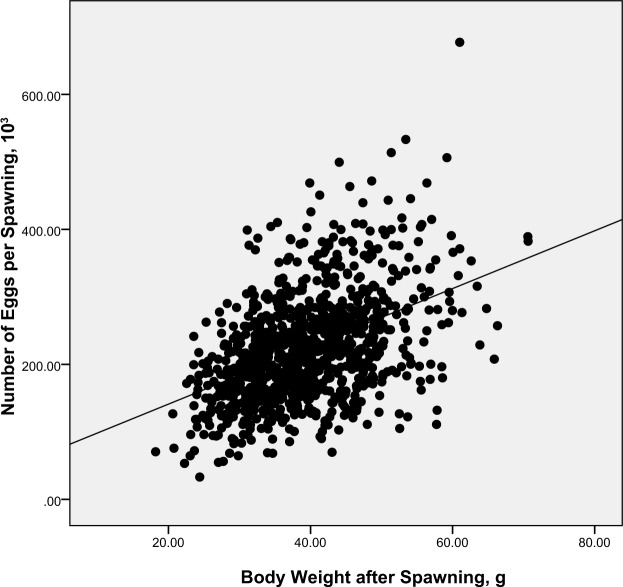
Phenotypic relationship between body weight after spawning (WAS) and number of eggs per spawn (NE), NE (10^3^) = 4.341 × WAS (g) + 54.427.

**Figure 2:**
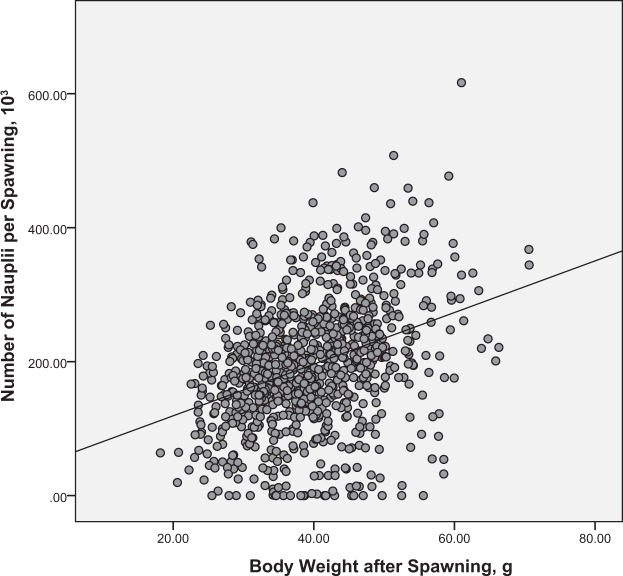
Phenotypic relationship between body weight after spawning (WAS) and number of nauplii per spawn (NN), NN (10^3^) = 3.964 × WAS (g) + 42.144.

Results of a linear regression analysis between WAS and NE are presented in Fig. 1. The phenotypic correlation between raw data of body weight after spawning (WAS) and number of eggs per spawn (NE) was moderate (*r* = *0.45* ± *0.03*) yet highly significantly different from zero (*P* < *0.01*). Similarly, the number of nauplii per spawn (NN) was also positively correlated with post-spawning weight (*r* = *0.35* ± *0.03, P* < *0.01* - Fig. 2).

### Frequency distribution of number of females spawning

**Figure 3:**
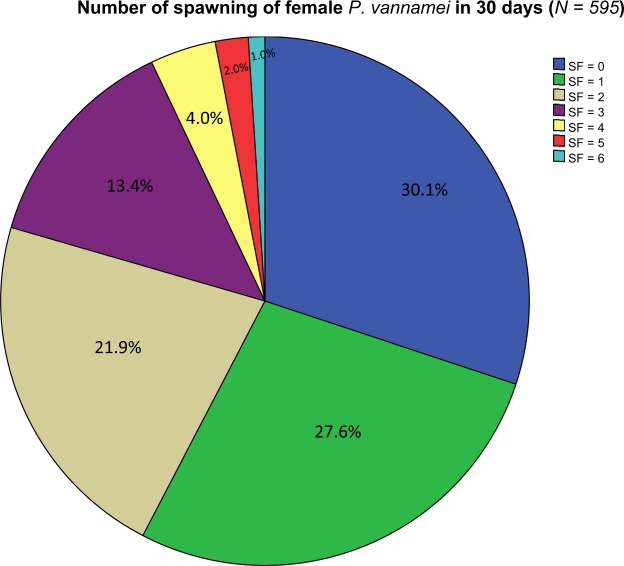
Number of spawns for 595 females over the 30 day trial (SF, spawning frequency).

Fig. 3 presents the information on the distribution of spawning records for 595 females. It is clear that about 30% of the females failed to spawn and 70% contributed with at least one spawning event over the trial period. 42.3% of females recorded two or more spawning events.

### Phenotypic and genetic variances among traits

Table 2 presents the estimated phenotypic and genetic variances and *h*^2^ values (Model 1) for each reproductive trait. *h*^2^ for body weight after spawning (WAS) was very high (0.64 ± 0.10), while *h*^2^ for the number of eggs per spawn (NE), number of nauplii per spawn (NN) and spawn frequency (SF) were all moderate (0.15 to 0.26). *h*^2^ estimates for hatching rate of eggs (HR and HR^at^) and the number of eggs per unit body weight (FE) were in general, low (0.04 ± 0.03) and not significantly different from zero. This result indicates that only limited additive genetic variance was present in the line for HR and FE traits. Estimates for repeatability variance and repeatability (*rep*) are presented in Table 2.

**Table 2:** Estimates of variance components, heritability values (*h*^2^) and repeatability (*rep*) for WAS, NE, NN, HR, HR^at^, FE and SF. *σ*^2^_*p*_, phenotypic variance; *σ*^2^_*a*_, additive genetic variance; *σ*^2^_*Ep*_, permanent environment variance.

Traits	Variance components			Heritability (±SE)	Repeatability (±SE)
	*σ*^2^_*p*_	*σ*^2^_*a*_	*σ*^2^_*Ep*_	*h*^2^	*rep*
WAS	60.13	38.47	3.15	0.64 ± 0.10^*^	0.69 ± 0.13^*^
NE	5735.74	1467.83	23.81	0.26 ± 0.07^*^	0.26 ± 0.07^*^
NN	7853.15	1384.53	101.17	0.18 ± 0.06^*^	0.19 ± 0.06^*^
HR	0.05	0.00	0.00	0.04 ± 0.03 ^NS^	0.04 ± 0.03 ^NS^
HR^at^	311.68	12.75	1.32	0.04 ± 0.03 ^NS^	0.04 ± 0.03 ^NS^
FE	2.85	0.14	0.00	0.05 ± 0.04 ^NS^	0.05 ± 0.04 ^NS^
SF	1.79	0.26	*NA*	0.15 ± 0.06^*^	*NA*

^*^Estimate is highly significantly different from zero (P < 0.01).

^NS^Estimate is not significantly different from zero (P > 0.05).

WAS, NE, NN, HR, HR^at^, FE and SF: see legend in Table 1.

*NA*, Not applicable.

### Genetic and phenotypic correlations among reproductive traits

Table 3 presents both genetic (*r*_*g*_) and phenotypic (*r*_*p*_) correlations between pairs of reproductive traits. The genetic correlations between WAS and reproductive traits (NE, NN, and SF) were high-medium, with 0.93 ± 0.10, 0.84 ± 0.10, and 0.57 ± 0.18, respectively. Genetic correlation between WAS and HR was low (0.02 ± 0.33) with large standard errors. However, a negative genetic correlation (−0.50 ± 0.27) was observed between WAS and FE, suggesting that while NE and NN will be increased with body weight, FE, the ratio of number of eggs per biomass, will be decreased. Regarding reproductive traits of NE, NN, and SF, these directly determine the production for nauplii hatcheries, and HR is an important index for egg quality. The above genetic relationships suggest that selection on fast growth will also benefit the seed sector, with no negative impact on egg quality. A negative genetic correlation between WAS and FE however, indicates that selection on fast growth may involve a trade-off in relative fecundity. Across all pairwise comparisons among reproductive traits, the highest genetic correlation were 0.97 ± 0.03 (NE and NN), 0.86 ± 0.18 (SF and NE), and 0.70 ± 0.24 (SF and NN), respectively.

**Table 3:** Estimated genetic (below diagonal) and phenotypic correlations (above diagonal) for body weight at spawning and reproductive traits^*^ (estimates ± se).

	WAS	NE	NN	HR	FE	SF
WAS		0.45 ± 0.03	0.35 ± 0.03	0.02 ± 0.04	−0.12 ± 0.09	0.23 ± 0.02
NE	0.93 ± 0.06		0.87 ± 0.01	0.23 ± 0.04	0.80 ± 0.02	0.10 ± 0.04
NN	0.84 ± 0.10	0.97 ± 0.03		0.65 ± 0.02	0.72 ± 0.02	0.14 ± 0.01
HR	0.02 ± 0.33	0.31 ± 0.37	0.55 ± 0.29		0.22 ± 0.04	0.12 ± 0.01
FE	−0.50 ± 0.27	−0.17 ± 0.42	−0.02 ± 0.42	0.40 ± 0.55		0.03 ± 0.03
SF	0.57 ± 0.18	0.86 ± 0.18	0.70 ± 0.24	−0.18 ± 0.45	0.44 ± 0.49	

^*^For abbreviations see legend in Table 1.

A similar pattern was evident for phenotypic correlations (*r*_*p*_) as that seen for genetic correlations among the traits evaluated. Except for the comparison of FE and WAS, all phenotypic correlations were positive, with the highest estimate evident between NE and NN and the lowest estimate observed between SF and FE. It is clear that the positive *r*_*g*_ estimates between WAS and reproductive traits were significantly higher than the corresponding *r*_*p*_ estimates.

## Discussion

The major difficulty associated with studying reproductive traits in penaeid species is establishing optimal environmental conditions for maturation over an experimental test period because the majority of reproductive traits tested are highly sensitive to even small variation in physical conditions; in maturation tanks, nutritional factors in broodstock diet and the physiological condition of experimental animals^5,32^. Across the 30 day experimental period in our trial, the majority of data on reproductive traits (Table 1) were very similar to those observed in the best performing Pacific white shrimp nauplii commercial hatcheries in China indicating in general, that our system for broodstock maturation and management was appropriate. While the accumulated spawning frequency (6.2%) in our study (percentage of females in the population spawning per night) was lower than in some high performing commercial nauplii hatcheries (optimal mating rate range is 12–15%)^33^, it was still within the range routinely observed under commercial conditions (5–12% per night)^34^. Our relatively low average accumulated matings per night may have resulted from a size effect of female broodstock used in the experiment as no body size selection of experimental animals was practiced here from PL to maturation stage. A large proportion of small sized female broodstock used here potentially have produced a relatively low spawning frequency compared with that possible if larger individuals had been chosen for the study specifically. While we observed this effect in our study (Ren *et al*., in press), positive correlations between body size of broodstock and spawning frequency have also been reported previously in a variety of farmed penaeid species^35,36^.

The mean number of eggs per spawn (NE) reported here (225.15 × 10^3^) was consistent with the estimates reported in two other studies on reproductive traits in female *P. vannamei* in Mexico^26,27^ (mean = 217.90 × 10^3^; 216.0 × 10^3^), and higher than the values (160.7 × 10^3^) reported by Tan, *et al*.^28^. The estimates of nauplii per spawn (NN) and hatching rate of eggs (HR) in our study were similar to the estimates reported by Arcos *et al*.^26^ (NN = 187.8 × 10^3^, HR = 86.20%), but significantly higher than that reported by Caballero-Zamora *et al*.^27^ (NN = 47.0 × 10^3^, HR = 21.76%) and Tan *et al*.^28^ (NN = 34.7 × 10^3^, HR = 21.59%), respectively. The different results likely arise from the differences in the mating design methods used for data collection between studies as the latter two studies employed artificial mating while our study and that of Arcos *et al*.^26^ relied on natural matings. Many factors, including physical condition of male/female broodstock or the artificial insemination (AI) skills of technicians, can significantly impact performance, which can lead to low hatching rates and a low number of nauplii produced. Because our experiment relied on natural matings, the conditions were very similar to practical commercial production conditions for nauplii production. We would expect therefore, that the data produced here would be more applicable to improving seed reproduction in industrial environments.

The distribution pattern of spawn frequency per female for 595 females (Fig. 3) was similar to that observed for nauplii production in most penaeid species where only a relatively small proportion of the mature female population are multiple spawners that contribute proportionally, the majority of nauplii produced in a spawning population^24,37–40^. Approximately a third of all of our females failed to spawn at all. Mean spawning frequency (SF) across the 30 day trial was 1.44, lower than reported in the study by Arcos *et al*.^26^ where mean SF was 1.71 per female per month. This difference may result from the different culture conditions used for broodstock in the two studies. In the Arcos *et al*.^26^ study, broodstock were cultured in an earthen pond while broodstock used in this study were cultured from PL to maturation stage in recirculating tanks. It is quite common for broodstock cultured in earthen ponds to show higher mean spawning frequency than broodstock cultured in tank systems. This effect was observed in our earlier study where we compared data on reproductive traits between broodstock reared in earthen ponds and recirculating tanks and confirmed the finding (Ren, *et al*., in press). In this comparison, mean SF for broodstock raised in earthen ponds was 1.93, a result similar to that reported by Arcos *et al*.^26^, but significantly higher than that of broodstock raised in recirculating tanks (SF = 1.34). While selecting for spawning frequency shows promise with a moderate degree of heritability (0.15), it should be recognised that very few females actually contribute to the next generation (see Fig. 3) and any future selection program should take this into account, to avoid potential negative impacts of inbreeding.

### Heritability estimates

*h*^2^ for body weight after spawning (WAS) was high (0.64 ± 0.10), and while higher than many reported for this trait in other penaeid species, it was still within the normal range. For Pacific white shrimp, mean *h*^2^ estimates for body weight during insemination are also high at 0.44 ± 0.08^27^, 0.49 ± 0.14^28^ and 0.58 ± 0.08^25^, respectively. While in black tiger shrimp, *h*^2^ for body weight at 54 weeks was 0.53 ± 0.14^31^. For the same trait in Nile tilapia, *h*^2^ was 0.68 ± 0.10^21^. *h*^2^ for body weight prior to spawning in red tilapia was higher 0.80 ± 0.16^20^. While levels of quantitative genetic variation for specific traits do vary among farmed populations and species (e.g. crustaceans vs fish), in general there are some consistencies^41^. In coho salmon, *h*^2^ estimates for both body weight at spawning and post-spawn weight were only moderate^15^. So comparatively, based on our estimates of Pacific white shrimp and the most recent published studies in most aquaculture species, body weight at spawning stage in most aquaculture species appears to be a highly heritable trait, indicating that a large amount of additive genetic variance is available that can be exploited in breed improvement programs in most aquatic species that have been tested in this way. Taken together, in general high heritability for weight/size at maturation stage provides good support for Hill’s (2010: p79) argument that ‘Heritabilities (*h*^2^) tend to be highest for conformation traits and mature size, typically 50 per cent or more, and lowest for fitness-associated traits such as fertility (Falconer and Mackay, 1996; Lynch and Walsh, 1998; Mousseau and Roff, 1987)’^41–44^.

The *h*^2^ estimate for SF was moderate (*h*^2^ = 0.15) and is similar to the estimates in earlier reports for this trait in *P. vannamei*^24^, but higher (*h*^2^ = 0.07) than a reported for females cultured in brackish water^25^. Moderate levels of additive genetic variation for SF indicate that multiple spawning capacity is an inherited trait and can therefore in theory, be improved via selection. *h*^2^ for NE (*h*^2^ = 0.26) and NN (*h*^2^ = 0.18) here were comparable with estimates reported for black tiger shrimp^31^, but higher than estimates reported in earlier Pacific white shrimp studies^26–28^. Differences between our estimates and those of Caballero-Zamora *et al*.^27^ and Tan *et al*.^28^ on the same species, once again, likely result from the different mating designs employed, in particular use of natural mating vs an artificial insemination approach. In salmonid species, *h*^2^ estimates for number of eggs and eyed eggs are usually moderate to high^14,15,19^. While in contrast, in tilapia, estimates for fecundity related traits are often quite low^20,21^. This however, is not always the case for farmed tilapia strains because number of eggs (*h*^2^ = 0.20) and number of hatched fry (*h*^2^ = 0.16) showed moderate heritability estimates in the GIFT tilapia strain^45^.

Our *h*^2^ estimate for relative fecundity, FE (*h*^2^ = 0.05), is the first report for this trait in a penaeid species. A low FE heritability estimate for Pacific white shrimp is consistent with estimates for this trait in Nile tilapia^21^. Likewise, nearly zero additive genetic variance was reported for a similar trait (fry/kg of broodstock) in channel catfish^23^. In general, low and non-significant additive genetic variance for FE traits suggest that relative reproductive output would most likely be a difficult trait to improve via genetic selection in most aquatic species.

*h*^2^ estimates for egg hatching rate (HR) estimated in our study were also low, a result consistent with similar estimates in both tilapia^20,21^ and channel catfish^23^. A related trait (number of larvae per female at hatching) reported for giant freshwater prawn also showed low additive genetic variance^46^. In general, traits that involve fertilization and/or survival of larvae at hatching are essentially group fitness traits rather than individual ones, indicating that potentially many non-genetic factors can have significant effects on such traits. Overall, limited additive genetic variation for HR indicates that this trait in Pacific white shrimp is unlikely to be improved via genetic selection.

### Genetic and phenotypic correlations

The estimated genetic correlations between body weight of WAS and fecundity traits (NE, NN, and SF) were all positive and medium-high in level (from 0.57 to 0.93), a result higher than earlier reported results for *P. vannamei*, e.g. correlations of 0.49 and 0.54 between body weight at insemination and NE, and NN, respectively^27^. Positive genetic correlations that are in general, moderate in degree between bodyweight at spawning and fecundity traits have also been reported in tilapia^21^ and some salmonids^14,15,19^. The genetic correlation between WAS and HR in our study was low and not significantly different from zero, which suggests that the genetic control of these two traits are not linked. Genetic correlations between WAS and reproductive traits of NE, NN, SF, and HR tested here were all positive, a result that suggests that selection to increase body weight of female broodstock will not have any negative impacts on their individual reproductive performance, and in fact could actually increase overall female broodstock reproductive output via indirect selection. In aquatic species, broodstock with relatively high body weight at spawning usually have experienced good nutrition and are likely to be in a robust physiological condition, as a consequence their fecundity should be higher than individuals that have experienced poorer nutrition. The genetic correlations results here for *P. vannamei* females however, suggest that high body weight and better reproductive performance are linked.

Genetic correlations among reproductive traits (NE, NN, HR, and SF) were all positive except for that of SF vs HR, and ranged from low (NE vs HR) to high (NE vs NN). These results show clearly that genetic selection on individual reproductive traits is unlikely to have any potentially negative impact on other reproductive traits. While this set of reproductive traits is very important for nauplii production in commercial Pacific white shrimp hatcheries, to date no published studies have reported on genetic correlations among these traits. Data from other studies have reported on correlations between some of these traits however, that have employed artificial insemination rather than natural matings in penaeids. As examples, the genetic correlation between NN and NE was reported to be moderate (0.24 ± 0.41) in *P. vannamei*^27^. For black tiger shrimp Macbeth *et al*.^31^, reported negative genetic correlations between NE and NN, and NE and HR, but moderate and positive correlations between NN and HR. In other aquaculture species, results of estimates for genetic correlations among reproductive traits have been very diverse^15,20,21^ and most may be species specific and will also depend on the specific pairs of traits examined.

The phenotypic correlation between WAS and NE was moderate (0.45), a result that is comparable with other estimates for *P. vannamei* e.g. *r*_*p*_ 0.34^26^ and 0.27^27^. A relatively high *r*_*p*_ between WAS and NE has also been reported in some other penaeid species^28,31^. The correlation between WAS and NN was lower than between WAS and NE but was still moderate and positive (see Figs. 1 and 2). A lower *r*_*p*_ for WAS and NN compared with that for WAS and NE may result from data with several spawning females showing a zero NN record; this phenomenon usually results from successfully mated females losing their adhered spermatophore during the spawning period. Comparisons of data from reproductive studies of penaeid taxa show that *P. vannamei* is included in a group of species with an open thelycum (a secondary sexual character involved in sperm transfer and storage in females), that have always been observed to show a small percentage of spawns that do not result in hatched nauplii. This results from otherwise successfully mated females losing their adhered spermatophore before mature eggs can be released. In this study, the phenotypic correlations between WAS and other reproductive traits were also all positive which indicate that larger females in general spawn more often, release a larger number of fertilized eggs and produce a larger number of nauplii than do smaller females^6,37^. Therefore as a general rule, large body size for mature females is a good practical and measurable characteristic for inferring likely relative reproductive performance of individual female *P. vannamei* broodstook.

### Implication for *P. vannamei* selection programs

Improving reproductive performance of Pacific white shrimp strains is a key objective for the broodstock market in China in the future. Fresh, nutritional food for broodstock maturation is a major financial constraint on hatcheries, and the nauplii production sector would benefit significantly from strains with improved reproductive output. This is because it would allow hatchery managers to maintain smaller broodstock populations while achieving comparable seed production output or allow them to invest the same resources but produce more seed. Both options would result in a significant increase in hatchery profit. The capacity to improve nauplii production from each female broodstock individual has huge commercial potential, with the possibility that spawning frequency could be trebled. Breeders can also include reproductive traits in addition to current selected traits via either a multiple trait selection approach or a two stage selection approach. Additionally, a genome selection (GS) approach has recently been very successful at improving total egg production in the poultry industry, so trialling GS in penaeid hatcheries may offer new potential for improving the reproductive traits in shrimps. Results may also be useful for genetic improvement of black tiger shrimp (*P. monodon*), as this species has a similar reproductive cycle to that of Pacific white shrimp and there are still major difficulties associated with nauplii production in black tiger shrimp farming.

## Conclusions

Results from the current study clearly demonstrate and confirm that additive genetic variation exists for an important set of female reproductive performance traits that include; number of eggs per spawn, number of nauplii per spawn and multiple spawning capacity in our domesticated Pacific white shrimp line in China and so these traits are likely to be improved via genetic selection. In contrast, limited additive genetic variation was also evident for some other reproductive performance traits notably: egg hatching rate and the relative fecundity per weight (g) of individual broodstock females, so these two latter traits are unlikely to be improved via genetic selection. Evidence for both positive and moderate genetic correlations and phenotypic correlations between body weight after spawning and some reproductive traits also suggests that there is only very limited potential for negative correlated impacts on individual reproductive performance of female broodstock by selection to increase mean body weight. It is clear however, that data will need to be collected over future multiple generations of selection from our domesticated line to investigate any potential for deterioration in reproduction performance due to the cooperative impacts of selection for improved mean body weight and accumulation of inbreeding over time.

## Materials and methods

### Experimental families

The experiment was conducted at a Beijing Shuishiji Biotech. Ltd. maturation facility in Wanning (Hainan Province), China. A base family line of *P. vannamei* was produced following a complete 4 × 4 diallel cross of four domesticated culture lines obtained in China between June and July 2015. Details of the strains and maintenance of families generated are described in Ren *et al*.^47^. On May 4^th^ 2016, when shrimp had reached maturation, 660 test animals were selected randomly from each of 78 full-sib families with individuals transferred to maturation tanks (10 m^2^). Test females were then subjected to unilateral eyestalk ablation to induce ovarian maturation, and broodstock males and females reared separately with 110 individuals per tank.

In total, data from 595 females that survived 30 days of experimentation were recorded in the study. Number-coded silicon eye rings were placed on the remaining eyestalk for individual female identification. A biological water recirculating system was used to maintain water quality at an exchange rate of 600% to 800% per day. Daily feed composition consisted of a mixture of fresh meal diet (50% polychaetes, 30% squid and 20% mussels) delivered at a rate of approximately 5% of total biomass per tank. Tank water was maintained at 28 ± 2 °C and 31–35 ppt salinity. Data collection commenced in June 2016 and continued over a 30 day period.

### Measurement of reproductive traits

Females presenting with mature ovaries (stage IV) were collected daily at 10:00 and transferred to tanks containing male broodstock. At 19:00, successfully-mated females with attached spermatophores were placed in individual 500 L fibreglass tanks filled with 300 L of clean seawater. Environmental conditions for spawning were maintained at 28 ± 0.5 °C and a salinity of 32–36 ppt. At 24:00, all females in spawning tanks were returned to maturation tanks and eggs incubated with gentle aeration.

Body weight after spawning (WAS) was measured after successful spawning at 12:00. Five reproductive traits were measured and recorded per female: (i) number of eggs (NE) per spawn - measured in three replicate 200 ml breaker samples after being thoroughly homogenized in seawater taken from spawning tanks (300 L in volume); (ii) number of nauplii (NN) per spawning - measured in the same way as for NE after nauplii had hatched on the second day at 11:30; (iii) relative fecundity - measured as the number of eggs per unit body weight (FE) - calculated by dividing the number of eggs per spawn by WAS; (iv) egg hatching rate per spawn (HR: the ratio between viable nauplii and total number of eggs per spawn); and v) female spawning frequency (SF) - calculated as the total number of successful spawning events per individual female at the end of the experimental period.

### Statistical analysis

Prior to formal data analysis, raw HR percentage data were arcsine transformed^48^. For comparative purposes, both the original data (HR) and arcsine transformed data (HR^at^) were used in subsequent statistical analyses. For all other traits (WAS, NE, NN, FE and SF), untransformed values were used in the data analysis. Genetic variance and covariance components and *h*^2^ for targeted traits were estimated using an animal model applying the restricted maximum likelihood (REML) methodology in WOMBAT^49^. For trait of SF, the linear mixed animal model can be written as follows:

1 $$y=X{\rm{\beta }}+Z{\rm{\alpha }}+e,$$

where:

***y*** is a vector of observations for a reproductive trait per spawn event (SF), ***β*** is a vector of fixed effects consisting of maturation tanks, the recorded spawn event batches, and regression coefficient of age of shrimps, ***α*** is the vector of random additive genetic effects of the animals, ***e*** is the vector of random residual errors and

***X*** and ***Z*** are known incidence matrices relating observations to the fixed effects mentioned above, and animal effects, respectively. Both *α* and ***e*** follow a normal distribution with mean zero and variance **A**σ_a_^2^ and **I**σ_e_^2^, respectively. Here, σ_a_^2^ and σ_e_^2^ are the additive genetic and residual error variances while **A** is the numerator relationship matrix based on pedigree information.

In this experiment, estimates of WAS, NE, NN, HR, HR^at^ and FE resulted from multiple observations for some female individuals. Repeated measures for these traits were across 30 days, with these data recorded from different spawning order, i.e. first spawn, second spawn, up to sixth spawn for some females. Therefore, we also used a repeatability animal model (Model 2) to account for replication within individuals:

2 $$y=X{\rm{\beta }}+{Z}_{1}{\rm{\alpha }}+{Z}_{2}pe+e,$$

where:

***y*** is a vector of observations for a reproductive trait per spawn event (WAS, NE, NN, HR, HR^at^, or FE), ***β*** is a vector of fixed effects consisting of maturation tanks, the recorded spawn event batches, and regression coefficient of age of shrimps, ***α*** is the vector of random additive genetic effects of the animals, ***pe*** is the vector of random maternal permanent environment effects contributed by individual females to their offspring families, ***e*** is the vector of random residual errors and ***X, Z***_***1***_ and ***Z***_**2**_ are known incidence matrices relating observations to the fixed effects mentioned above, animal effects and permanent environment effects, respectively. **α, pe** and ***e*** follow a normal distribution with mean zero and variance **A**σ_a_^2^, **I**σ_pe_^2^ and **I**σ_e_^2^, respectively. Here, σ_a_^2^, σ_pe_^2^ and σ_e_^2^ are the additive genetic, permanent environment and error variances while **A** is the numerator relationship matrix based on pedigree information.

In Model (1), *h*^2^ was calculated as the ratio of additive genetic variance to total phenotype variance (*h*^2^ = σ_α_^2^/ σ_p_^2^). In Model (2), heritability (*h*^2^) and repeatability (*rep*) were calculated as follows:

$${h}^{2}=\frac{{{\rm{\sigma }}}_{a}^{2}}{{{\rm{\sigma }}}_{a}^{2}+{{\rm{\sigma }}}_{pe}^{2}+{{\rm{\sigma }}}_{e}^{2}},\,rep=\frac{{{\rm{\sigma }}}_{a}^{2}+{{\rm{\sigma }}}_{pe}^{2}}{{{\rm{\sigma }}}_{a}^{2}+{{\rm{\sigma }}}_{pe}^{2}+{{\rm{\sigma }}}_{e}^{2}}$$

In the primary analysis, the results were similar between the linear mixed animal model and the repeatability animal model, which were also supported by comparing the log-likelihood ratio of the models. Therefore, a multivariate mixed animal model was fitted to estimate the genetic and phenotype correlation between examined traits, expressed in matrix notation as:

3 $$[\begin{array}{c}{\rm{yWAS}}\\ {\rm{yNE}}\\ {\rm{yNN}}\\ {\rm{yHR}}\\ {\rm{yFE}}\\ {\rm{ySF}}\end{array}]={\boldsymbol{X}}{\boldsymbol{\beta }}+{\boldsymbol{Z}}{\boldsymbol{\alpha }}+{\boldsymbol{e}},$$

where **yWAS**, **yNE**, **yNN**, **yHR**, **yFE** and **ySF** are the same as defined in Model 1, respectively. Total phenotypic variance (σ_p_^2^) was estimated as the sum of additive animal genetic variance (σ_α_^2^) and random residual components (σ_e_^2^). Genetic or phenotypic correlation between two traits was calculated as: $r=\frac{{\sigma }_{12}}{\sqrt{{\sigma }_{1\,}^{2}}\sqrt{{\sigma }_{2\,}^{2}\,}}$ where σ_12_ was the genetic or phenotypic covariance between two traits, and σ_1_^2^ and σ_2_^2^ were either additive genetic variances of trait 1 and 2, or phenotypic variances of the two traits, respectively.
